# The Center for Epidemiologic Studies Depression Scale is an adequate screening instrument for depression and anxiety disorder in adults with congential heart disease

**DOI:** 10.1186/s12955-017-0747-0

**Published:** 2017-09-05

**Authors:** Ju Ryoung Moon, June Huh, Jinyoung Song, I-Seok Kang, Seung Woo Park, Sung-A Chang, Ji-Hyuk Yang, Tae-Gook Jun

**Affiliations:** 1 Department of Nursing, Grown-Up Congenital Heart Clinic, Heart Vascular and Stroke Institute, Samsung Medical Center, Sungkyunkwan University School of Medicine, Seoul, Korea; 2 Department of Pediatrics, Grown-Up Congenital Heart Clinic, Heart Vascular and Stroke Institute, Samsung Medical Center, Sungkyunkwan University School of Medicine, Seoul, Korea; 3 Division of Cardiology, Grown-Up Congenital Heart Clinic, Heart Vascular and Stroke Institute, Samsung Medical Center, Sungkyunkwan University School of Medicine, Seoul, Korea; 4 Department of Thoracic & Cardiovascular Surgery, Grown-Up Congenital Heart Clinic, Heart Vascular and Stroke Institute, Samsung Medical Center, Sungkyunkwan University School of Medicine, Seoul, Korea

**Keywords:** Depression, Anxiety, Screening, Congenital heart disease

## Abstract

**Background:**

The Center for Epidemiological Studies Depression Scale (CES-D) is an instrument that is commonly used to screen for depression in patients with chronic disease, but the characteristics of the CES-D in adults with congenital heart disease (CHD) have not yet been studied. The aim of this study was to investigate the criterion validities and the predictive powers of the CES-D for depression and anxiety disorders in adults with CHD.

**Methods:**

Two hundred patients were screened with the CES-D and secondarily interviewed with a diagnostic instrument, i.e., the Mini International Neuropsychiatric Instrument. The sensitivity and specificity values of the CES-D were calculated by cross-tabulation at different cutoff scores. Receiver operating characteristic (ROC) curves were used to assess the optimal cutoff point for each disorder and to assess the predictive power of the instrument.

**Results:**

The CES-D exhibited satisfactory criterion validities for depression and for all combinations of depression and/or anxiety. With a desired sensitivity of at least 80%, the optimal cutoff scores were 18. The predictive power of the CES-D in the patients was best for major depression and dysthymia (area under the ROC curve: 0.92) followed by the score for any combination of depression and/or anxiety (0.88).

**Conclusion:**

The use of CES-D to simultaneously screen for both depression and anxiety disorders may be useful in adults with CHD. Trial registration: CESDEP 212. Registered 2 March 2014 (retrospectively registered).

## Background

As a result of recent advancements in cardiac surgery, more than 85% of patients with congenital heart disease (CHD) reach adulthood, and 200,000 such patients are estimated to have reached adulthood in Korea [1]. However, among this population, more than 55% have presented with medical problems such as arrhythmia, bacterial endocarditis, congestive heart failure, and pulmonary vascular diseases, and have needed subsequent operations even after receiving childhood surgery [2]. Many adults with CHD also suffer from various psychological difficulties caused by the heart disease itself, e.g., fear of death, treatment decision-making, anxiety associated with the preparation for cardiac surgeries, maladjustment to implanted cardiac devices, and the transition from childhood to adulthood [3, 4]. With regards to their emotional functioning, there is an inconsistency in the literature. Some studies have reported that when compared with control groups [5, 6], adolescents and adults with CHD have poorer emotional functioning. However, other studies have reported that adolescents and adults with CHD fare well [7]. The inconsistency in findings could be attributed to the use of different measurement tools. Therefore, evaluation using the same tool with CHD-specific should be performed, and development of an intervention to increase emotional health should be based on these results [7, 8].

In Korea, awareness of the psychological issues associated with CHD in adults has increased, and research in this field has recently been active [3, 9–11]. However, research on the development of psychological issues and related factors in adults with CHD in Korea, which differs from other countries in terms of social, economic, and cultural characteristics, is lacking. To be more specific, Westerners are reared to grow up as an independent and autonomous human being. However, Koreans are raised differently, affected by Confucianism and a collectivist culture centered around the family. The Korean culture emphasizes the absence of a self-concept, a social order that always prioritizes the group over the individual, the trait of saving one’s face as seen in shame culture, control based on excessive power and authority in patriarchal family systems, rigid gender roles, and a characteristic parent-child relationship [12]. Therefore, the Western psychosocial evaluation criteria may not apply to Koreans, who have a different social and cultural environment.

Moreover, in Korea, where the degree of understanding and empathy for psychiatric illnesses is low and psychiatrists are not as accessible, it is critical that cardiologists who have already formed a rapport with patients to identify those with psychological issues. Such identification would improve the psychological care provided to these patients.

The screening tools used to investigate the psychological problems (i.e., depression and anxiety) that create great discomfort and difficulties for adults with CHD [7, 13] are, in fact, not used to definitively diagnose depression and anxiety. However, they can still improve diagnostic efficiency by helping to identify patients likely to have such psychological problems. Moreover, it is imperative that the psychological screening tools are well matched to those that are used officially by psychiatrists.

The Center for Epidemiological Studies Depression Scale (CES-D) is used clinically as a screening tool for various patient groups [14] because it has excellent sensitivity and reliability as a tool for diagnosing depression [15–17]. However, there are currently no reported cases of this scale being applied to adults with CHD. The CES-D cutoff scores differ depending on patient group and are higher for groups with more psychiatric risk factors than control groups [15]. Thus, if the CES-D is to be applied to adults with CHD, it is necessary to investigate an optimal cutoff score.

Anxiety and depression are highly interrelated and share overlapping symptoms. Therefore, the CES-D can be used to evaluate anxiety as well as depression [18, 19]. Simultaneous screening for both depression and anxiety with a single tool represents advantages for both the examiners and patients because it is more convenient and time-efficient. However, the evaluation of anxiety in adults with CHD with the CES-D has not yet been reported. Further, the cutoff score used to diagnose anxiety may differ from that used for depression.

The purpose of this study was to evaluate the criterion validity of the CES-D as a screening test for the diagnosis of anxiety and depression in adults with CHD and to determine appropriate CES-D cutoff scores for depression and anxiety.

## Methods

### Materials and procedures

This was a prospective study of patients with CHD from the outpatients of the Grown-Up Congenital Heart (GUCH) Clinic, which is single tertiary center at the Samsung Medical Center. The criteria for selection were as follows: 1) age greater than 18 years, 2) absence of complications or syndromes associated with severe intellectual handicap(s) (e.g., trisomy 21), 3) the ability to understand and complete the questionnaires, and 4) Willingness to participate in the survey. The study period ranged from November 2013 to May 2014. In total, 212 patients visited the Samsung Medical GUCH clinic during this period. We excluded 12 patients; 6 had been diagnosed with Marfan syndrome and the remaining responded inadequately to the survey questions. Thus, the final analysis included 200 patients. The sample size satisfied the requirements for the test of the validity of the questionnaire [20, 21].

The survey was conducted after approval of the study protocol was obtained from the Samsung Medical Center Institutional Review Board. If the patients agreed to participate in the study, they were asked to sign a consent form and complete a questionnaire. One cardiovascular outpatient nurse collected the survey data (i.e., the CES-D, a questionnaire about functional status and demographic data) during one-to-one interviews during the patients’ visits to the outpatient clinic for check-ups or tests.

One-hundred twelve patients who scored higher than 9 for depression on the CES-D were recruited for a face-to-face interview, the Mini International Neuropsychiatric Instrument (MINI) by a single psychologist on the same day. To investigate the patients’ clinical characteristics, a researcher collected and reviewed the electronic medical records.

### Instruments

#### CES-d

The symptoms of depression and anxiety were assessed with the CES-D, which was developed by Radolff [22] and translated into Korean by Nam and Lee [23]. The CES-D was designed specifically to screen for depressive symptoms in the general population and in patients with chronic disease [14], but it has also been used to screen for anxiety symptoms [19, 24]. The CES-D is a self-reported tool consisting of 20 items including four statements that are rated on a scale of 0 to 3. The patients select a rating to describe how they felt during the previous week. The Korean version of the CES-D has adequate test/retest reliability (0.68 over several weeks), internal consistency (0.89–0.93) [23], and concurrent validity and requires approximately 4–5 min to complete. Scores range from 0 (lowest) to 60 (highest), and patients are categorized into one of the following four groups: a) not depressed (0–9 points), b) mildly depressed (10–15 points), c) moderately depressed (16–24 points), or d) severely depressed (more than 25 points). The standard cutoff point of 16 or more was used to classify patients with depressive symptoms [25]. The internal consistency of the CES-D score in this study was 0.92.

#### Mini international neuropsychiatric interview

To test the criterion validity of the CES-D, the Mini International Neuropsychiatric Interview (MINI) was used. The MINI, which was developed by Sheehan et al. in 1997, is a short, structured diagnostic interview for major axis I diseases according to the Diagnostic and Statistical Manual of Mental Disorders, 4th edition and the International Statistical Classification of Disease and Related Health Problems, 10th revision [26]. The MINI can diagnose major depression, dysthymia, panic disorder, social phobia, agoraphobia, general anxiety disorder (GAD), and other coexistent disorders and can be administered within 15 min. Structured psychiatric interviews, which take less time but produce a high level of accuracy, are required in epidemiological studies and multicenter clinical trials and to track outcomes in non-research clinical settings. In this regard, the MINI satisfies the conditions required for such structured psychiatric interviews. According to standardized data collected from 270 patients at 10 university hospitals and psychiatry clinics, the current internal consistencies of the questions for each psychiatric diagnosis, with the exception of drug abuse, are 0.60–0.84, and the test-retest reliability is excellent (above 0.75) [27].

#### Sociodemographic and clinical characteristics

The sociodemographic characteristics of the participants included age, gender, education level, occupation, marital status, and monthly average household income. Household income was classified into the following three categories: low class (under 15.00 million won), middle class (15.00 to 41.59 million won), and high class (over 41.60 million won) [28–30]. The clinical characteristics of CHD diagnosis and frequency of cardiac surgeries were investigated, and functional classes were investigated using the New York Heart Association (NYHA) functional classification [31] and percutaneous oxygen saturation (SaO_2_) level.

### Statistical analysis

SPSS Statistics version 22.0 for Windows (SPSS Inc., Chicago, IL) was used for the data analysis. The general clinical characteristics were analyzed according to the real numbers, percentages, means, and standard deviations. The sensitivity, specificity, and positive predictive values for depression and anxiety of the CES-D were calculated by cross-tabulation and receiver operating characteristics (ROC) for multiple CES-D cutoff scores (i.e., 16, 18, 20, and 22). The specificities of the CES-D for the selected depressive and anxiety disorders, positive predictive value (PPV), and negative predictive value (NPV) were also analyzed by cross-tabulation and ROC. The associations between the CES-D scores and the diagnostic measurements were calculated using ROC curves. Finally, to investigate the possibility of a difference in the patterns of the two scores, we compared the mean scores for the 20 items of the CES-D for depression and for anxiety disorders.

## Results

### Demographic and clinical characteristics of the participants

The demographic and clinical characteristics of the participants in this study are summarized in Table 1. The average age of the participants was 38.7 years, and 45% were female. Half (50%) lived alone, 53% had completed primary or secondary education, and 47% had completed higher or scientific education. Almost 70% (69.2%) had a job, and 49% were in the middle-income category (Table 1). In terms of CHD diagnoses, 36.5% of the participants had atrial septal defects, 13.5% of the subjects had ventricular septal defects, and 31.5% had a diagnosis of cyanotic heart disease. Over 45% of the participants were in each of the NYHA functional classes I and II, 38% had undergone one cardiac operation, 24.3% had undergone two cardiac operations, and 22.1% had undergone three or more cardiac operations. More than three-quarters (77.2%) of the participants had an oxygen saturation (SaO_2_) level greater than 95, and 11.3% had an SaO_2_ level less than 90% (Table 1).

**Table 1:** Demographic & clinical characteristics of participants (*N* = 200)

Characteristics	Categories	N(%) or Mean ± SD
Gender	Male	90(45.0)
Age(year)		38.7 ± 12.9
Education level	High school ≥ College	94(53.0) 106(47.0)
Occupation	Yes	138(69.2)
Marital status	Single Married Divorced/Widowed	78(39.1) 100(50.0) 22(10.9)
Monthly expenditure (10,000 won)	Low (<150) Middle (150–415) High (≥416)	42(21.3) 98(49.0) 57(28.7)
Primary CHD^*^ diagnosis	***Acyanotic CHD*** ASD VSD Valvular disease (AR, MR) PDA Ebstein’s anomaly L-TGA complete AVSD	***137(68.5)*** 73(36.5) 27(13.5) 15(7.5) 9(4.5) 6(3.0) 4(2.0) 3(1.5)
	***Cyanotic CHD*** TOF Eisenmenger syndrome PA with VSD DORV D-TGA PA	***62(31.5)*** 23(11.5) 16(8.0) 12(6.0) 6(3.0) 3(1.5) 2(1.0)
NYHA functional class	Class I Class II Class III Class IV	91(45.5) 91(45.5) 18(9.0) 0(0.0)
Number of cardiac operation	0 1 2 3 4	31(15.6) 76(38.0) 49(24.3) 28(14.0) 16(8.1)
Percutaneous SaO2^†^	<85 85 ~ 90 91 ~ 95 ≤96	11(5.3) 12(6.0) 23(11.5) 154(77.2)

The values are expressed as mean ± standard deviation; and qualitative variables, as percentages of the total

*Abbreviations*: *CHD* Congenital Heart Disease, *ASD* Atrial septal defect, *VSD* Ventricular septal defect, *AR* Aortic regurgitation, *MR* Mitral valve regurgitation, *PDA* Patent Ductus Arteriosus, *L-TGA* Levo-looped transposition of the great arteries, *AVSD* Arioventricular septal defect, *TOF* Tetralogy of Fallot, *PA* Pulmonary atresia, *DORV* Double outlet right ventricle, *D-TGA* Dextro-transposition of the great arteries, *NYHA* New York Heart Association, *SaO*_2_ arterial oxygen saturation

The subjects’ depression scores ranged from 0 to 53 with a mean of 18.4 (SD 5.9). A total of 14% of the participants were suffering from severe depression, and 28.5% of the participants had depression and/or anxiety (Table 2).

**Table 2:** Descriptive statistic of the questionnaire used in this study (*N* = 200)

Variables	Categories	N(%) or Mean ± SD
CES-D score		***18.4 ± 5.9***
	Not depressed(0–9)	88(44.0)
	Mildly depressed(10–15)	47(23.5)
	Moderate depressed(16–24)	37(18.5)
	Severe depressed(≥25)	28(14.0)
MINI;		
Depressed and/or anxiety		57(28.5)
Major depressive disorder		31(15.5)
Generalized anxiety		20(10.0)
Dysthymia		8(4.0)
Panic disorder		4(2.0)
Social phobia		4(2.0)
Agoraphobia		4(2.0)
Depressive disorder^a^		39(19.5)
Anxiety disorder^b^		36(16.0)
Depressive disorder^a^ and co-morbid anxiety disorder^b^		12(6.5)

The values are expressed as mean ± standard deviation; and qualitative variables, as percentages of the total. *MINI* Mini international neuropsychiatric interview

^a^major depressive disorder and dysthymia

^b^one or more of the following disorders: generalized anxiety, panic disorder, social phobia, agoraphobia

*Abbreviations*: *CES-D* Center for Epidemiologic Studies Depression Scale, *MINI* The Mini-International Neuropsychiatirc Interview

### Sensitivity, specificity, and positive predictive value of the CES-D for depressive and anxiety disorder

The sensitivities, specificities, and PPVs results from the MINI for different cutoff points are presented in Table 3. When the aims were to detect major depressive disorder (MDD) (sensitivity, 84.6%; specificity, 65.3%) and dysthymia (sensitivity, 73.2%; specificity, 60.3%) together, the best cutoff point was 18. When screening for anxiety disorders, the sensitivities for the detections of a GAD and for all anxiety disorders together were optimal at the cutoff point of 18 (GAD, 87.3%; all anxiety disorders, 84.7%). Additionally, the specificity levels for GAD and all anxiety disorders together at this cutoff point were 70.5 and 72.4%, respectively. When the CES-D was used to screen for any depressive and/or anxiety disorder using the cutoff point of 18, the PPV doubled from 28.5% at baseline to 53.6%. The NPV remained above 90% for all of the various disorders at different cutoff points with the exception of anxiety disorders at the cutoff point of 20 (NPV = 89%; NPV not shown).

**Table 3:** Sensitivity, specificity a positive predictive value of the CES-D for depressive and anxiety disorder

	CES-D ≥ 16			CES-D ≥ 18			CES-D ≥ 20			CES-D ≥ 22		
	Sens(%)	Spec(%)	PPV(%)	Sens(%)	Spec(%)	PPV(%)	Sens(%)	Spec(%)	PPV(%)	Sens(%)	Spec(%)	PPV(%)
DAD	91.4	56.6	24.4	83.9	80.4	53.6	72.3	79.6	50.3	66.0	88.7	59.6
MDD	86.7	61.8	39.0	84.6	65.3	28.6	88.0	83.4	43.2	80.3	79.5	40.2
GAD	88.1	59.4	28.1	87.3	70.5	15.3	77.8	73.5	16.7	65.9	77.9	18.9
DYS	88.1	57.4	12.4	73.2	60.3	6.5	71.4	69.3	6.9	56.7	77.3	6.3
DEP	71.4	52.3	4.9	85.6	79.2	34.0	73.3	75.1	24.6	60.0	76.3	24.3
ANX	91.2	56.3	19.1	84.7	72.4	28.9	80.0	78.9	36.8	78.2	85.7	48.2

*Abbreviations*: *DAD* depression and/or anxiety disorder, *MDD* major depressive disorder, *GAD* generalized anxiety disorder, *DYS* dysthymia, *DEP* depressive disorder, *MDD & DYS*, *ANX* anxiety disorder: panic disorder, phobia, generalized anxiety disorder, *Sens* sensitivity, *Spec* specificity, *PPV* positive predictive value

The predictive power of the CES-D in these participants was best for MDD and for depressive disorders, i.e., the sum of the MDD and dysthymia scores. The AUCs as identified with ROC analysis were 0.92 for MDD (standard error (SE) = 0.029; 95% confidence interval (CI) 0.83–0.95; *p* < 0.001) and depressive disorder (SE = 0.031; 95% CI 0.82–0.92; *p* < 0.001). The second best predictive power was observed for depressive and/or anxiety disorder for which the AUC was 0.88 (SE = 0.031; 95% CI 0.83–0.91; *p* < 0.001). The predictive power for GAD was also very good at 0.85 (SE = 0.028; 95% CI 0.79–0.93; *p* < 0.001).

### Differences between depression and anxiety scores

The patterns of scores for each of the 20 CES-D items individually did not differ between depressive and anxiety disorders (Table 4).

**Table 4:** Mean scores of CES-D items concerning depressive and anxiety disorders

CES-D items(range 0: rare ~3: most)	Depressive disorder	Anxiety disorder	Difference(BI)
	Mean (SD)	Mean(SD)	
I was bothered by things that don’t usually bother me	0.8(0.8)	0.7(1.0)	0.1(−0.8–0.6)
I didn’t feel like eating	0.6(0.7)	0.5(0.8)	0.1(−0.8–0.4)
I felt that I could not shake off the blues	0.7(0.8)	0.5(0.7)	0.2(−0.6–0.3)
I felt that I was just as good as other people	1.5(1.0)	1.4(0.8)	0.1(−0.4–0.8)
I had trouble keeping my mind on what I was dong	0.8(0.8)	0.6(0.5)	0.2(−0.8–0.3)
I felt depressed	1.3(0.8)	1.2(0.7)	0.1(−0.5–0.6)
I felt everything I did was an effort	1.3(1.0)	1.4(1.1)	0.1(−0.4–0.4)
I felt hopeful about the future	1.5(1.0)	1.3(0.9)	0.2(−0.8–0.6)
I thought my life had been a failure	0.9(1.0)	1.0(1.0)	0.1(−0.7–0.6)
I felt fearful	0.7(1.0)	0.8(0.9)	0.1(−0.4–0.7)
My sleep was restless	1.1(0.9)	1.2(1.2)	0.1(−1.0–0.3)
I was happy	1.7(1.0)	1.5(1.1)	0.3(−0.4–0.8)
I talked less than usual	1.0(1.0)	1.1(0.8)	0.1(−0.5–0.4)
I felt lonely	1.1(1.0)	1.2(1.0)	0.2(−0.7–0.3)
People were unfriendly	0.7(0.8)	0.2(0.7)	0.5(−1.0–0.3)
I enjoyed life	1.5(0.8)	1.3(1.0)	0.2(−0.4–0.4)
I had crying spells	0.7(0.8)	0.9(1.1)	0.2(−0.5–0.8)
I felt sad	0.8(0.9)	0.6(0.8)	0.2(−0.5–0.4)
I felt that people disliked me	0.5(0.7)	0.7(0.4)	0.4(−0.6–0.4)
I could not get ‘going’	0.7(0.9)	0.9(0.7)	0.2(−0.5–0.5)

## Discussion

This study was conducted to examine the CES-D and its validity as a screening tool for psychological symptoms such as depression and anxiety in adults with CHD, as well as to determine its diagnostic cutoff points.

Approximately 28–35% of adults with CHD experience depression [12, 32]. The selection of patients who are at a high risk of psychological problems in the early stages and their referral to psychologists or psychiatrists reduces the incidence of more serious psychological disorders. This study verified that the CES-D is appropriate as a screening tool for the early stages of anxiety and depression in adults with CHD.

The CES-D was originally developed to evaluate depressive symptoms in general population epidemiological studies [22] and is currently widely used in various clinical settings as a case-finding measure for depression and as stand-alone diagnostic instrument [14]. It is commonly used to screen and detect major depression. No significant differences have been found between different clinical settings [14].

In clinical settings, the Patient Health Questionnaire (PHQ-9) is also used to detect major depression. It is often used as an alternative to the CES-D and has been used commonly in many clinical settings. The PHQ-9 evaluates depression symptoms and severity [14]. The PHQ-9 has excellent diagnostic accuracy, a sensitivity of 0.80 (95% CI: 0.71–0.87) and a specificity of 0.92 (95% CI: 0.88–0.95) [33]. When the PHQ-9 and the CES-D were compared with respect to their positive likelihood ratios, 22–35% of non-patients were diagnosed with a mental disorder with the CES-D [33] compared to 53% with the PHQ-9 [14]. Therefore, the CES-D is more suitable than the PHQ-9 for use as a tool for screening for depression.

It is best to attain both high sensitivity and specificity when determining a cutoff point. However, in the case that this may be impossible, it is more important to choose high sensitivity over specificity if the instrument is used for screening over diagnosis [14]. With regard to sensitivity, the CES-D exhibited similarly excellent results for depressive disorders and anxiety disorders. These results are consistent with those obtained following the application of the CES-D to elderly patients living in residential homes [24]. When investigating depression and anxiety together by setting the cutoff point at the score of 18, the predictive power of the tool was also satisfactory. In other words, the criterion validities were satisfactory and occurred in the following descending order: when only depression disorder was present, when both depression and anxiety disorders were present, and when only anxiety disorder was present.

In this study, the cutoff points were the same for depression and anxiety. The cutoff points for MDD and/or dysthymia were 18, whereas this value increased to 20 when only MDD was present. This value was higher than the cutoff point of 16 that was set at the time of the development of the CES-D [25] and is similar [17] to that recommended after a systematic meta-analytic review that was conducted to identify an appropriate cutoff point for the CES-D as a screening tool [14]. Several questions in the CES-D address physical symptoms. Thus, the score can easily be higher for patients with poor functional class due to heart and lung function, as is often for adults with CHD [9]. It is necessary to raise the cutoff point for the optimal screening of patients with heart or lung disease, including CHD. Overall, screening for depression and anxiety with the CES-D tool can be useful in the clinical setting as it is both convenient and efficient.

The CES-D can also be used practically during regular screenings because it has maximal sensitivity to identify depression and minimize missed cases [14]. Minimizing the false-positive rate is vital, especially in Korea where psychiatric resources are limited and psychiatry is generally misunderstood. This can be achieved by increasing the cutoff point.

On the contrary, when the cutoff point is increased, a greater number of cases may be missed. When the CES-D is used for screening, the probability of correctly identifying depression and anxiety increases two-fold at the individual level. The probability of correctly identifying at the cutoff scores for all psychiatric disorders is greater than 90%, indicating satisfactory levels of identification [14, 24]. However, the CES-D is not recommended for use as an isolated diagnostic tool given the results that show low positive likelihood ratio. Therefore, further studies are needed to verify diagnoses made with the CES-D [14, 24].

Additionally, because the participants were not randomly selected, there could have been a referral bias. Therefore, participants who were concerned about their mental health or already had depression may have participated in our study. Such participants may have overstated their symptoms, and diagnosis of depression may therefore have been overestimated. Therefore, a well-structured randomized study will be needed in the future.

In conclusion, the CES-D was used as an initial screening tool to identify participants who need further in-depth assessment of their depressive symptoms. However, it is not recommended as a stand-alone diagnostic tool. Therefore, additional diagnostic evaluations are still required for all participants with scores greater than 18. Specifically, follow-up diagnostic evaluations are needed to confirm the diagnoses of disorders and also to distinguish depressive disorders and anxiety disorders.

## Conclusion

The CES-D is highly accurate as a screening tool for adults with CHD in clinical settings; however, the interpretation of the results requires attention because there is the possibility that scores above 18 indicate depressive disorders as well as anxiety disorders. The CES-D can be used to screen for both depression and anxiety disorders.

## Abbreviations

ACHD: Adults with congenital heart disease
AUC: Area under curve
CES-D: Center for Epidemiological Studies Depression Scale
CHD: Congenital heart disease
CI: Confidence interval
GAD: General anxiety disorder
GUCH: Grown-Up Congenital Heart
MDD: Major depressive disorder
MINI: Mini International Neuropsychiatric Instrument
NPV: Negative predictive value
NYHA: New York Heart Association
PPV: Positive predictive value
ROC: Receiver operating characteristic
SaO_2_: Oxygen saturation
SE: Standard error
